# MSAIRHD: Mainland Southeast Asia International River Upper Reach Hydrological Dataset, 1958–1987

**DOI:** 10.1038/s41597-026-07860-6

**Published:** 2026-07-11

**Authors:** Qingping Cheng, Xuefu Pu, Zhenzhou Zhang

**Affiliations:** College of Soil and Water Conservation, Southwest Forestry University, Kunming, Yunnan 650224 China

## Abstract

Southeast Asia’s international rivers sustain the livelihoods of hundreds of millions of people and represent globally important biodiversity hotspots and ecologically sensitive regions. The scarcity of hydrological station data has constrained our understanding of long-term hydrological evolution, water cycle dynamics, and hydrological modelling in these basins. Here, we present a compilation of historical daily records of streamflow (87 stations), sediment concentration (40 stations), and water level (118 stations) in the upper reaches of the Irrawaddy, Salween, Mekong, and Red River basins for the period 1958–1987. Based on these records, we derived 74 hydrological indices. For streamflow, data were available from 12 stations in the Irrawaddy basin (mean record length 23 years), 13 in the Salween basin (18.8 years), 34 in the Mekong basin (21.1 years), and 28 in the Red River basin (23.3 years). Water level records covered 14 Irrawaddy stations (mean 20.92 years), 20 Salween stations (15.57 years), 48 Mekong stations (18.75 years), and 35 Red River stations (19.68 years). Sediment concentration data were available for 5 Irrawaddy stations (mean 13 years), 7 Salween stations (15.85 years), 16 Mekong stations (14.37 years), and 12 Red River stations (17.25 years). Overall, water level stations are the most numerous across the four basins, whereas sediment concentration stations are the fewest. The derived hydrological indices are crucial for elucidating the historical hydrological evolution of the upper Irrawaddy, Salween, Mekong, and Red River basins. In addition, these data provide valuable support for interdisciplinary studies of transboundary river environments, hydrology, ecology, and natural flow reconstruction, and for calibrating and validating numerical models. This dataset fills a key gap in large-sample global hydrological studies by improving the representation of this previously underrepresented region.

## Background & Summary

Rivers are vital to human societies and natural systems, as they support livelihoods, socioeconomic development, ecosystems, human life and activities, and infrastructure, while also sustaining native biodiversity, ecosystem integrity, and biogeochemical cycles^1–5^. Global river streamflow observations are essential for advancing large-scale hydrological science and modeling^6,7^. However, the current lack of comprehensive streamflow sampling limits our ability to understand the substantial changes occurring within river systems^2,8^. High-quality streamflow datasets are also critical for improving our understanding and prediction of streamflow processes^9^ and for a wide range of socioeconomic and ecological analyses and applications. Yet, a high-resolution global streamflow dataset remains unavailable^8,10^. This gap constrains effective water resources management and limits the in-depth and accurate assessment of consequential changes in river systems^2^, including hydrological cycle processes, aquatic ecosystem functioning, and the development of hydrological science, particularly in transboundary river basins. For example, the Mekong River, a transboundary basin supporting the livelihoods of more than 70 million people and diverse terrestrial and aquatic ecosystems, is constrained by the lack of sufficient, reliable, and accessible observational data^11^. There is therefore an urgent need to collect and organize high-resolution hydrological data in international river basins, especially in the upper reaches of transboundary rivers such as the Irrawaddy, Salween, Mekong, and Red rivers.

In recent decades, the integration of remote sensing and observational data has enabled the development of numerous meteorological and hydrological data products at global and regional scales using statistical and machine learning approaches. These products include basin-scale physiographic attributes^12–15^, runoff datasets^7,10,16,17^, hydroclimatic data and indices^6,18–22^, and comprehensive datasets containing hydrometeorological variables, watershed characteristics, landscape information, and other related data^9,11,23–31^. However, these data products and indices remain severely limited by the scarcity of hydrometeorological stations, particularly in the upstream areas of several international rivers in southwestern China, thereby hindering the development of hydrometeorological science and modeling in this region. Large-scale hydrological analyses generally require extensive gauge-based hydrological datasets^19^ (the global indices do not include the Mainland Southeast Asia International River Upper Reach Hydrological Dataset), both globally and regionally. Rivers in Southeast Asia are important transboundary systems that support water supply, fisheries, irrigated agriculture, energy generation, riverine ecosystems, navigation, and recreation. However, as noted by *Tiwari et al.*^11^ in their study of the Lancang–Mekong River, data limitations have hindered more comprehensive basin-scale studies of rapidly changing hydrological, agricultural, and ecological systems. In particular, the Mekong River Basin continues to face major challenges due to very limited, discontinuous, sparse, or inaccessible observational datasets.

To our knowledge, studies based on hydrological station observations in the transboundary basins of the Dulong Jiang–Irrawaddy, Nu Jiang–Salween, Lancang Jiang–Mekong, and Yuan Jiang–Red River systems in Southeast Asia have mainly focused on analyses of the mainstream at annual, monthly, and daily scales^32–37^. To date, no observational dataset of multi-station hydrological regime indices has been released for the upstream reaches that covers observations of streamflow, sediment, and water level in both mainstreams and tributaries. The only existing products are streamflow datasets derived from multi-source data fusion constrained by satellite gravimetry^38^ and reservoir water storage data^39^. While observed streamflow, sediment load, and water level data are provided by *Tiwari et al*.^11^, these are limited to the lower Mekong River Basin, except for four streamflow stations in the upper Lancang River (Changdu, Gajiu, Jiuzhou, and Yunjinghong). In this study, we compiled high-quality hydrological records from the upper reaches of the Irrawaddy, Salween, Mekong, and Red River basins, sourced from the Hydrological Yearbooks published by the Ministry of Water Resources of the People’s Republic of China (covering the period 1958–1987, except 1986). These records underwent systematic quality-control procedures. Based on the quality-controlled records, we developed a suite of hydrological indices for the four upper transboundary river basins. Owing to data-sharing restrictions imposed by the data providers, the original station observations cannot be disseminated. The following sections describe the methods, data records, technical validation, and usage notes.

## Methods

### Study area

**Fig. 1:**
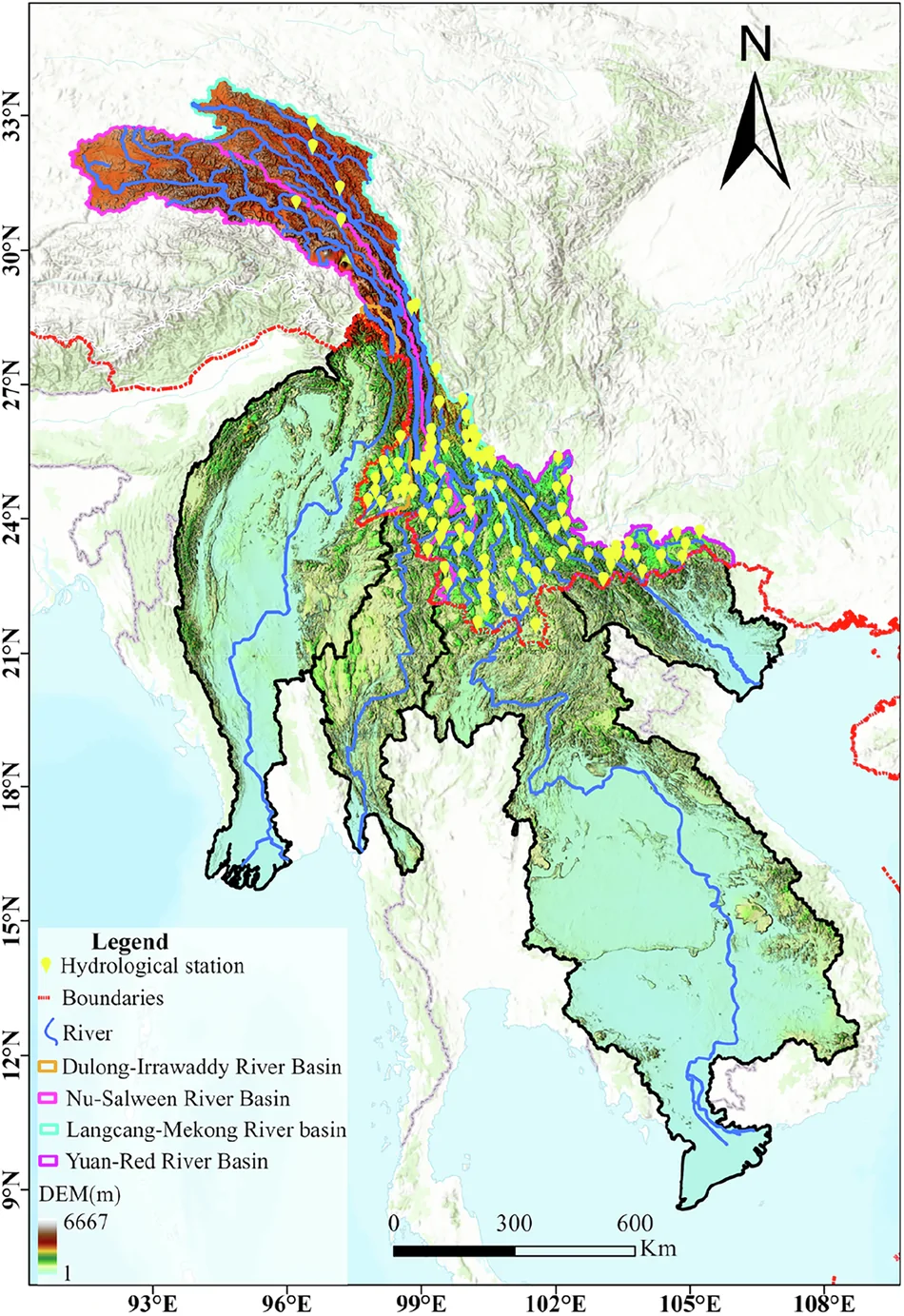
Information on hydrological stations and topographic maps in the study area.

This study selected the upper reaches of international rivers in southeastern China, including the Dulong Jiang (Irrawaddy River), Nu Jiang (Salween River), Lancang Jiang (Mekong River), and Yuan Jiang (Red River), as the research subjects (Fig. 1). The Dulong Jiang-Irrawaddy River originates in the southern margin of the eastern Himalayan Syntaxis. It runs for about 2,000 km, spanning the whole length of Myanmar and forming a large delta distributary network in the south prior to discharging into the Andaman Sea, with a basin surface area of 437,000 km^2^^40^. Irrawaddy River majority of the basin is situated in Myanmar, with small portions in China (5%) and India (4%)^41,42^. Originating from the Tanggula Mountains on the Tibetan Plateau, the Nu Jiang-Salween River flows through China, Thailand, and Myanmar before emptying into the Andaman Sea in the Indian Ocean. In China, it is referred to as the Nu Jiang, while the subsequent sections are known as the Salween River. The river has a total length of 3200 km and covers a basin area of 325,000 km^2,^^43^. The Lancang Jiang-Mekong River from the Tanggula Mountains on the Qinghai-Tibet Plateau in China and flows through six countries (i.e., China, Myanmar, Laos, Thailand, Cambodia, and Vietnam) before discharging to the South China Sea^44,45^. Being the largest transboundary river in Asia, it boasts a drainage area of 79.5 × 10^4^ km^2^ and stretches for a total length of 4880 km. The mean discharge at the estuary is approximately 4750 × 10^8^ m^3^, with an average flow rate of 15,060 m^3^/s^46^. The annual precipitation in the region is around 1370 mm^44,46^. The Lancang Jiang in China spans a length of 2161 km, covering a basin area of 16.5 × 10^4^  km^2^, which accounts for 20.7% of the overall basin area. The river maintains an average elevation of 3058 m, with its northern segment positioned on the Tibetan Plateau at altitudes exceeding 4000 m^44^. By 2021, a total of 103 hydropower reservoirs (China has already built 11 reservoirs on the main stem of the Lancang Jiang mostly for hydropower generation, with a total capacity of 42.17 km^3^) were operational in Lancang Jiang -Mekong River, boasting a combined installed capacity of 33 GW and a collective storage capacity of 100 km^3^^46,47^. The Red Jiang is situated in the Longitudinal Range-Gorge Region of Southwest China. It is a significant international river in China and Southeast Asia, traversing China, Vietnam, and Laos. The basin covers approximately 7.4 × 10^4^  km^2^, with a main channel of 677.0 km in China^48^.

### Daily hydrological data

This study collected data on streamflow, sediment concentration, and water level from the basins of the Dulong Jiang, Nu Jiang, Lancang Jiang, and Yuan Jiang. The data is sourced from International Hydrological Yearbook of Tibet and Yunnan in the People’s Republic of China, specifically the hydrological data for international rivers in Tibet and Yunnan (with missing streamflow data for 1986 and sediment concentration data missing for both 1973 and 1986). A total of 129 stations are situated along the mainstream and major tributaries, referred to as mainstream control stations and regional representative stations, respectively, to monitor the most significant hydrological variations. These include 87 streamflow stations, 40 sediment stations, and 118 water level stations. Quality control was conducted on the daily hydrological data for each river basin at each station. To maximize the retention of hydrological stations, we retained periods of at least 1 consecutive year of daily hydrological data. The observed streamflow, data underwent rigorous quality control and homogeneity testing to ensure high reliability. Consistent with *Wang et al*.^39^, streamflow inhomogeneities arising from station relocation, instrumentation changes, river diversions, and recalibration of rating curves were meticulously addressed using local expertise, historical records, and statistical methods. Furthermore, to mitigate digital errors resulting from data extraction from hydrological yearbooks, we conducted thorough and repetitive manual proofreading to ensure data quality.

### Hydrological indices

A total of 74 hydrological indices were computed using Python for *International_River_Hydrological_Indices.py* (https://github.com/Chengqp2022/International_River_Hydrological_Indices), covering streamflow (m^3^/s), sediment concentrations (kg/m^3^), and water level (m). These indices characterize key attributes including magnitude, frequency, duration, rate of change, timing, variability, and recession processes at annual and multi-annual scales. Detailed definitions of each index are provided in *Chen et al*.^19^ and are not repeated here. Table 1 lists some information about the relevant indices. It should be specially noted that sediment concentrations (kg/m³) and water level (m) in Table 1 do not include the indices Qhigh, Qlow, RM, BM, FreH, FreL, FreZ, NumH, NumL, NumZ, DurH, DurL, DurZ, HFD and BFI.

**Table 1:** Hydrometric indices in the MSAIRHD.

Category	Index name	Units	Definition
Magnitude	Qmax1, Qmax3, Qmax7, Qmax30, Qmax90,	m^3^ s^−1^	Maximums of consecutive 1, 3, 7, 30, and 90 d streamflow averages.
	Qmax1p, Qmax3p, Qmax7p, Qmax30p,	m^3^ s^−1^	percentages of the maximum of consecutive 1, 3, 7, and 30 d streamflow accumulation amounts, which are themaximums divided by the annual streamflow accumulation amounts and then multiplied by 100.
	Qmin1, Qmin3 Qmin7, Qmin30, Qmin90,		Minimums of consecutive 1, 3, 7, 30, and 90 d streamflow averages.
	Q1st, Q5th, Q10th, Q25th, Q50th, Q75th, Q90th, Q95th, Q99th	m^3^ s^−1^	The 1st, 5th, 10th, 25th, 50th 75th, 90th, 95th, and 99th percentiles of daily streamflow.
	Qmean1, Qmean2, Qmean3, Qmean4, Qmean5, Qmean6, Qmean7, Qmean8, Qmean9, Qmean10,Qmean11, Qmean12, Qmean	m^3^ s^−1^	Monthly and annual mean flows.
	Qhigh, Qlow	m^3^ s^−1^	High- and low-flow event thresholds. QHigh equals 9 times Q50th.
	RM, BM		Runoff magnitude and baseflow magnitude. RM and BM are the differences between the maximum and minimum of streamflow and baseflow respectively.
Frequency	FreH, FreL, FreZ		Frequencies of high-flow (FreH), low-flow (FreL), and zero-flow (FreZ) days.
	Fre1st, Fre5th, Fre95th, Fre99th		Frequencies of days with streamflow bigger than or smaller than thresholds of the 1st, 5th, 95th, and 99^th^ streamflow percentiles.
	NumH, NumL, NumZ		Numbers of streamflow events with thresholds of Qhigh, Qlow, and zero.
	Num1st, Num5th, Num95th, Num99th		Numbers of streamflow events with thresholds of the 1st, 5th, 95th, and 99th percentile of the whole multiyear streamflow time series.
Duration	DurH, DurL,DurZ, Dur1st,	days	Mean duration of streamflow events with thresholds of Qhigh, Qlow, and zero.
	Dur5th, Dur95th,Dur99th	days	Mean duration of streamflow events with thresholds of the 1st, 5th, 95th, and 99th percentiles of the whole multiyear. streamflow time series.
Changing rate	RBFI		Richards–Baker flashiness index.
	RRmean, RRmedian,FRmean, FRmedian	m^3^ s^−1^	RRmean and RRmedian are the mean and median of all positive differences between consecutive daily streamflow values in a year; FRmean and FRmedian are the mean and median of all negative differences between consecutive daily streamflow values in a year.
Timing	HFD	days	Half flow date (HFD) is the date on which half of a water year’s total streamflow has passed since start of thewater year.
	MMD	days since1 January	Momentary maximum date (MMD) is the date when the maximum streamflow occurs.
	MC7FD	days since1 January	Minimum consecutive 7 d flow date (MC7FD) is the date when the minimum of consecutive 7 d flow averages occurs.
Variability	VY		Variance of daily streamflow.
	COVY		Coefficient of variation of daily streamflow.
	QCV		QCV means quartile-based coefficient of variation of daily streamflow, which is calculated as (Q75th–Q25th)/Q50th.
	RMM		Ratio of Qmax1 to Q50th.
Recession	BFI		Baseflow index (BFI) is the ratio of baseflow volume to streamflow volume over a specific time period.

Note: for more sources and definitions of the indices, see *Chen et al*.^19^.

## Data Records

The MSAIRHD dataset has been published on Science Data Bank^49^ and includes three sub-datasets: (1) “MSAIRHD_station_catalogue.xlsx”, (2) “International_River_Hydrological_Indices.xlsx”, and (3) “Available station counts and years statistics.xlsx”. The “MSAIRHD_station_catalogue” dataset provides 74 indices for 129 stations, covering streamflow, sediment, and water level (with “Y” marked in the corresponding columns to indicate the availability of streamflow, sediment, and water level data for each station). In addition, this file includes mainstream river names, tributary river names, station names, station ID, longitude, latitude, basin area, and whether the station has been relocated (marked with “*”), and other information. The “International_River_Hydrological_Indices” file contains the specific indices for each station. The “Available station counts and years statistics” file summarizes the number of stations and the years of available observations for each station and each basin. Based on this information, users can select hydrological stations that meet their specific research requirements, thereby supporting a variety of analytical purposes.

## Technical Validation

**Fig. 2:**
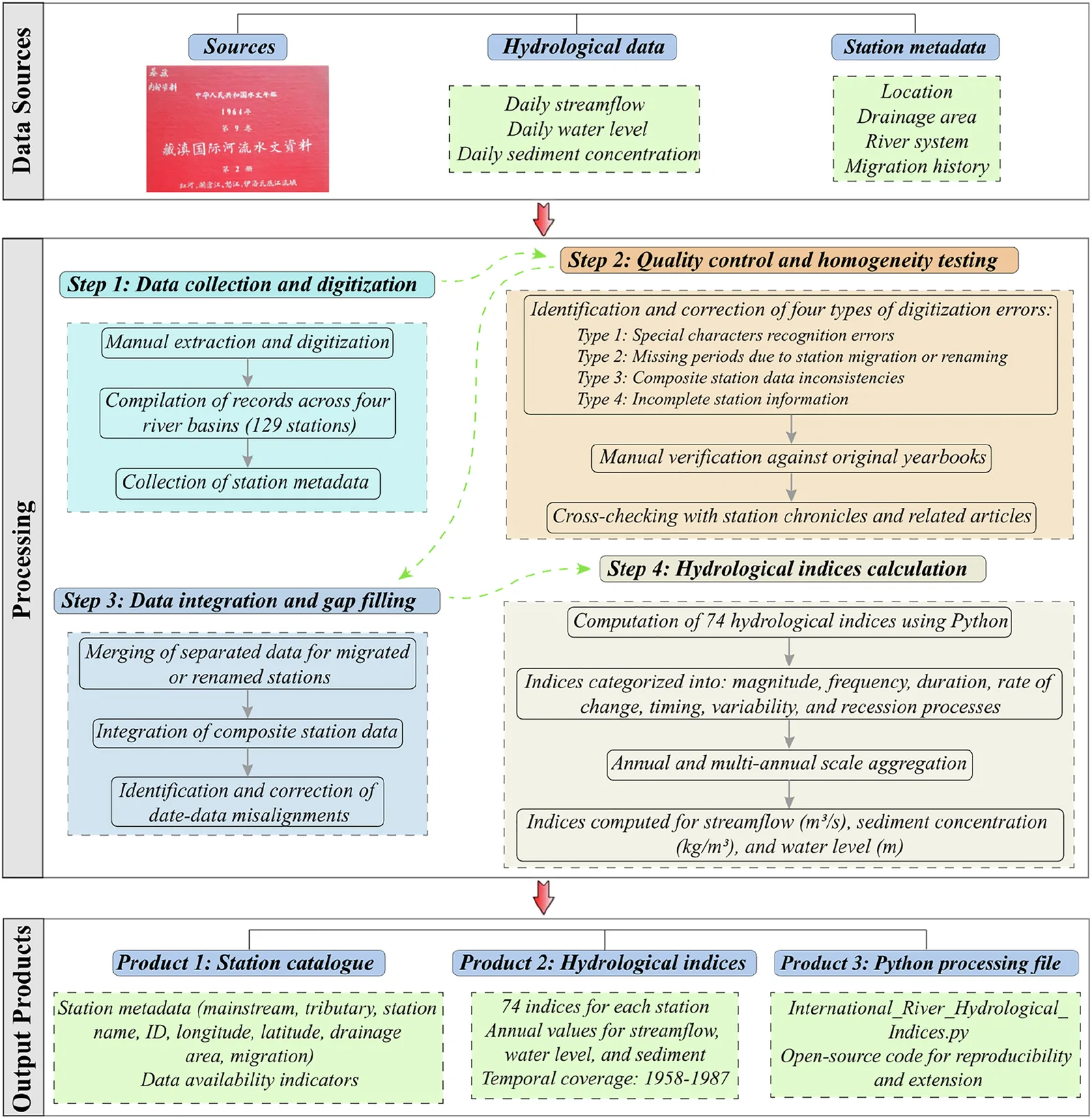
Framework of the methodology adopted in MSAIRHD for deriving the hydrological dataset and catalogue for the upper reaches of the Irrawaddy, Salween, Mekong, and Red River basins.

The data are sourced from the International Hydrological Yearbook of Tibet and Yunnan in the People’s Republic of China. Specifically, flow velocity measurements are routinely conducted at designated cross-sections using either propeller-type current meters or acoustic Doppler current profilers. These measurements are used to calibrate and frequently update site-specific stage–discharge rating curves, thereby converting daily stage records into mean discharge values. Suspended sediment concentration is determined through laboratory analysis of water samples collected with depth-integrating samplers in combination with cross-sectional sampling^39,50–53^. The hydrological measurements used in this study strictly comply with the national standards established by the Ministry of Water Resources (http://xxzx.mwr.gov.cn/xxgk/gbjb/zghlnsgb/), with the uncertainty in sediment flux measurements controlled within 10%^50^. Data extracted from the relevant hydrological yearbooks have been widely used in previous studies^39,50–53^. In addition, we validated the hydrological data used in this study through multiple approaches to ensure the reliability and accuracy of the MSAIRHD dataset. The specific technical processing workflow is shown in Fig. 2.

### Data quality assurance

The data for this research was primarily obtained through manual extraction and digitization. We conducted data digitization, proofreading, and correction of possible errors from April 5, 2023, to August 18, 2024. The quality control process for MSAIRHD followed a rigorous two-stage approach, adapted from established methodologies in large-sample hydrology studies. The main issues encountered during the data visualization study can be classified into several types:

***Type 1***. There are special characters and errors in the scanned extraction of yearbook data, such as: (1) 2 and 3 being recognized as 7, 8, and?, 0 being recognized as o, 8 or 96 being recognized as %, confusion between 0 and null values, and decimal points being recognized as colons; (2) some stations have errors in recognizing the last few days of December.

*Solution.* Cross-check and correct using hydrological yearbooks, seek missing data by re-scanning the yearbook, and manually verify recognition errors and special characters.

***Type 2***. Due to uncontrollable factors, there are cases of hydrological station migration or renaming, leading to missing periods in the data time series.

*Solution.* Manually merge data that should belong to the same hydrological station but are separated, based on latitude and longitude (within the same river or administrative area, such as counties and townships) or by checking the history of hydrological stations, station chronicles, and related articles.

***Type 3***. During data merging, the following situations may arise:

(1) Migrated or renamed stations may have duplicate data within the same year, where previous stations show null values for a few months, while the migrated or renamed stations show null values a few months before.

*Solution*. Merge by visual interpretation for unification.

(2) Some years directly provide composite hydrological data, while others have split composite stations into channels, etc.

*Solution.* For years without composite data, determine rivers, channels, etc., as the same hydrological station data according to the hydrological yearbook, and merge the corresponding river and channel data.

(3) There may be misalignment between dates and data during manual merging.

*Solution.* Verify against the hydrological yearbook and re-merge.

(4) Errors may occur in Python merging when different stations are mistakenly combined due to similar station names (e.g., the Luoluo Jiang of Huangping station and Luoluo Jiang of Huangping (Composite) Station; the Yalong Jiang of Ganzi station and Yalong Jiang of Huning Station).

*Solution*. Cross-check with the hydrological yearbook and manually correct errors.

***Type 4***. The compiled station information table lacks completeness in terms of river systems and latitude and longitude information; some migrated stations have different watershed areas across years.

*Solution*. Search for and add relevant river system and latitude/longitude information using the names of hydrological stations and their histories. For migrated stations, if the watershed area changes, further confirm if they belong to the same station. If they do, and the area change is minimal, take the latest station’s watershed area as the standard.

*Stage 2*. Time series reliability assessment.

We assessed the overall reliability of each hydrological time series based on:

*Data completeness*. Records with at least 1 consecutive year of daily hydrological data were retained to maximize station coverage while ensuring minimum data quality.

*Homogeneity testing*. Streamflow, sediment concentrations and water level inhomogeneities arising from station relocation, instrumentation changes, river diversions, and recalibration of rating curves were meticulously addressed using station chronicles, historical records.

**Fig. 3:**
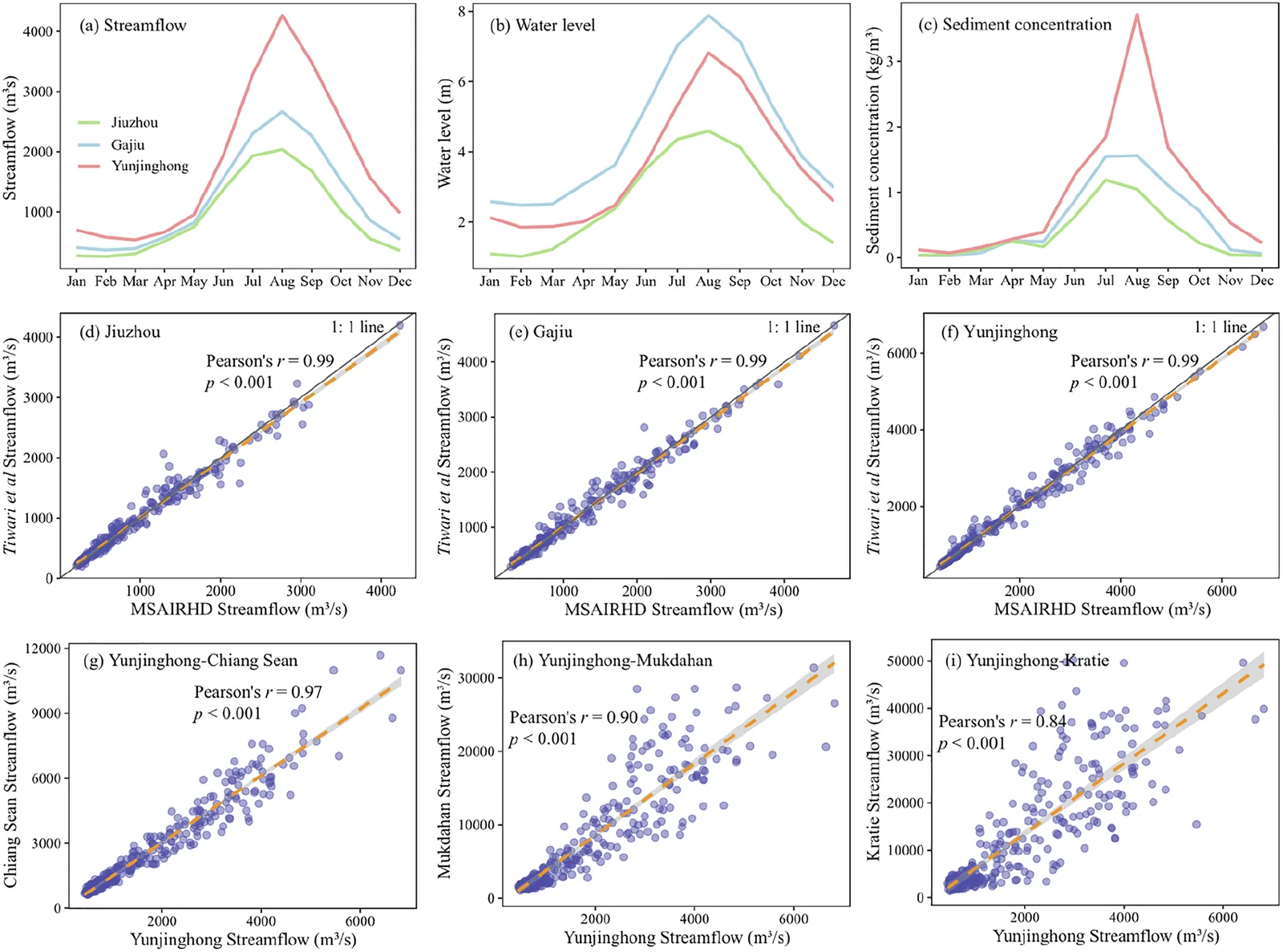
Monthly mean values of streamflow (m³/s), water level (m), and sediment concentration (kg/m³) at three representative stations in the upper (Jiuzhou), middle (Gajiu), and lower (Yunjinghong) reaches of the Lancang River Basin (a, b, c), and validation results of runoff between the three representative MSAIRHD stations and *Tiwari et al.*^11^.

*Spatial consistency*. We examined the spatial and temporal coherence of hydrological records across stations within the same river basin. Upstream-downstream relationships were verified to ensure that streamflow and sediment concentrations decreased upstream and increased downstream, following natural hydrological principles (Fig. 3).

*Seasonal patterns*. Validated that peak streamflow, sediment concentrations and water level occur during the monsoon season, coinciding with higher streamflow and erosion potential. (Fig. 3).

*Cross-validation with related hydrological variables*. Streamflow, sediment concentrations and water level were cross-checked to ensure physical consistency, as high flows typically correspond to elevated water levels and increased sediment concentrations. The seasonal mean water level at Yunjinghong being lower than at Gajiu may be related to downstream backwater effects or greater local inflow in the middle reaches. Overall, the variations in monthly mean streamflow, water level, and sediment load are physically consistent (Fig. 3).

*Validation against previously available data*. After validating the 1961–1985 annual mean streamflow results at the three representative upstream Lancang River stations previously reported by *Tiwari et al.*^11^, we found complete agreement with their results. The correlation between the Lancang River outlet station, Yunjinghong, and the three representative Mekong River stations was also relatively consistent, with the lowest *R²* values all exceeding 0.8. These findings indicate that changes in the upstream hydrological regime during the natural period were well correlated with downstream conditions, suggesting that changes in the Lancang River hydrological regime had a pronounced impact on the Mekong River.

### Summary of technical validation

The multi-level validation approach, combining manual quality control, inter-station consistency analysis, and comparison with available regional data, confirms the reliability of the MSAIRHD dataset. While some limitations exist due to data gaps and the scarcity of independent reference datasets for this region, the dataset represents the most comprehensive compilation of hydrological observations for the upper reaches of these transboundary rivers. Users are encouraged to consult the quality flags and completeness metrics provided in the station catalogue when selecting data for specific applications.

## Usage Notes

The Methods and Data Records sections provide a comprehensive overview for readers seeking to understand how the metrics were derived and organized. This will facilitate the informed use of the dataset in their research endeavors.

### Data selection and application guidelines

#### Station selection criteria

We highly recommend that users select hydrologic stations that contain data representative of the period of their study. The available time periods of observed streamflow data vary at different hydrological stations across the four basins. To facilitate station selection, the MSAIRHD_station_catalogue includes: station metadata (mainstream river names, tributary river names, station names, IDs, coordinates, drainage areas, streamflow, water level, sediment; Table 2); record length and temporal coverage; information on station migration and data gaps (see “Available station counts and years statistics.xlsx”).

**Table 2:** Station basic information.

Field	Description
1	Mainstream river names
2	Tributary river names
3	Station names
4	Number ID
5	Longitude
6	Latitude
7	AREA_skm
8	Migration
9	Streamflow
10	Water level
11	Sediment

Note: An asterisk (*) indicates the presence of station relocation. The “Y” in the columns for streamflow, hydrology, and sediment indicates the presence of corresponding data.

### Data quality considerations

**Fig. 4:**
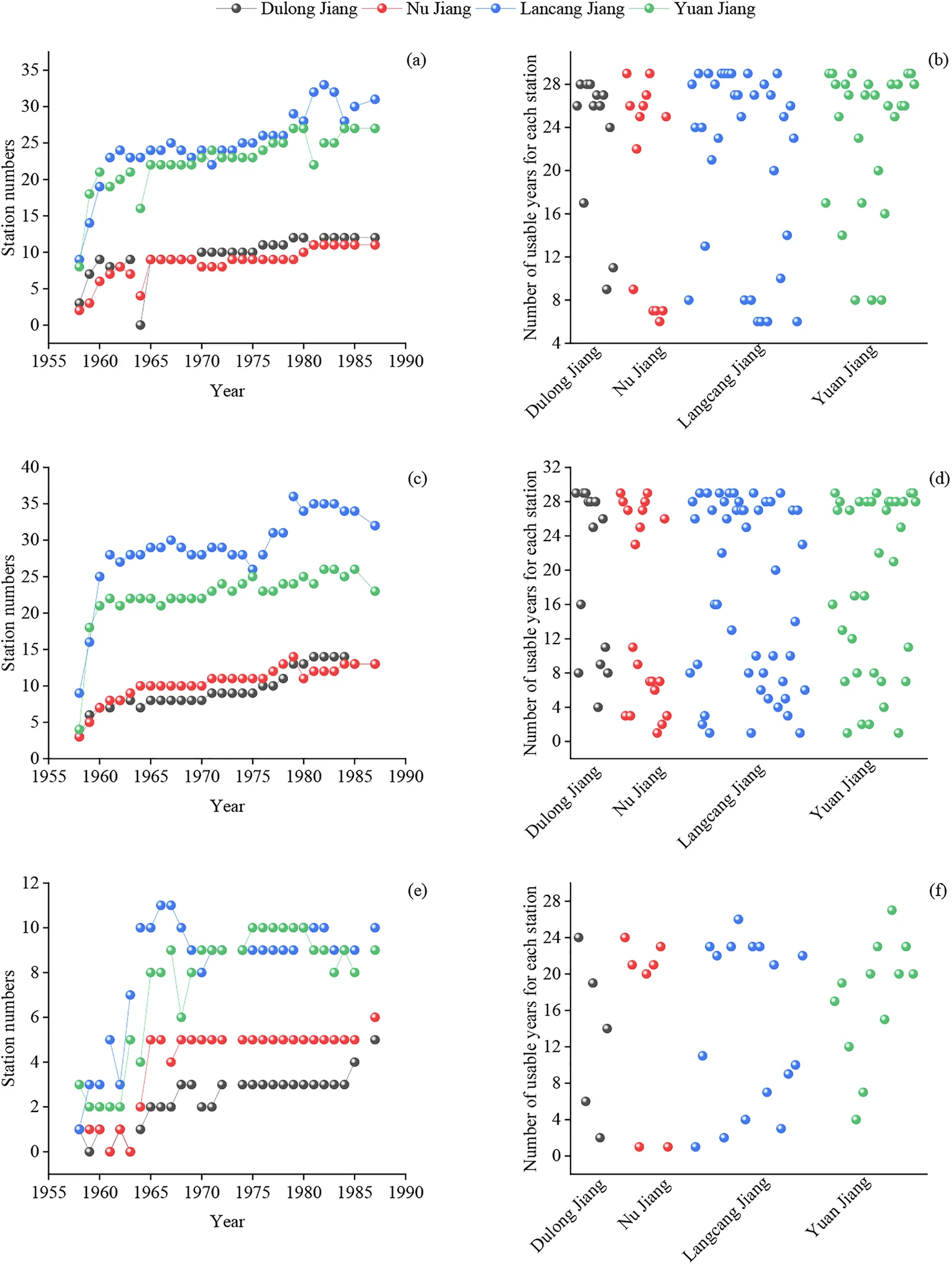
The number of available monitoring stations for hydrological indices (**a,b** for streamflow, **c,d** for water level, and **e,f** for sediment concentrations) each year and number of usable years for each station in each basin.

The dataset includes quality flags and completeness metrics that users should consider when selecting stations for analysis. Specifically:

Stations with longer records (≥20 years) provide more robust estimates of hydrological indices; the presence of gaps or missing years should be accounted for in trend analyses; users requiring high-frequency analysis should note that sediment concentration data is generally more limited than streamflow and water level records (Fig. 4).

### Spatial and temporal scope

The MSAIRHD dataset covers the period 1958–1987 (except 1986), representing a critical baseline period for understanding hydrological conditions prior to major dam construction and land use changes in the region. Users should be aware that: the dataset captures the upper reaches of these international rivers within Chinese territory; this period predates the construction of the majority of hydropower dams on the Lancang-Mekong mainstem; the dataset can serve as a reference for assessing subsequent hydrological changes.

### Recommended research applications

We envision the new dataset generated in this study can be applied in a wide range of research areas, including but not limited to:

#### Hydrological model calibration and validation

The MSAIRHD dataset provides crucial ground-truth data for calibrating and validating process-based hydrological models in a region where observational data has been historically scarce. The streamflow, water level, and sediment indices can be used to evaluate model performance for simulating: Daily and monthly discharge patterns; flow duration curves and extreme flow statistics; sediment transport dynamics; Seasonal flow regimes.

#### Climate change impact assessment

The long-term records (1958–1987) provide a unique baseline for investigating climate variability and the potential impacts of climate change on hydrological systems in this data-scarce region. These records enable researchers to: analyze trends in streamflow characteristics over the 30-year period; examine the relationship between climate variability and hydrological responses; and compare pre-development hydrological conditions with the current hydrological regime.

#### Transboundary water resources management

As an international river basin dataset, MSAIRHD supports:

Understanding upstream hydrological contributions to downstream countries; water availability assessments for the lower Mekong, Salween, Irrawaddy, and Red River basins; cross-border water resource planning and management.

#### Ecological and environmental applications

The suite of hydrological indices derived in this study (74 indices covering magnitude, frequency, duration, timing, and rate of change) can be used to: assess environmental flow requirements for river ecosystems; evaluate ecological responses to hydrological variability; support biodiversity conservation planning in these globally significant freshwater ecosystems; analyze habitat suitability for native fish species and aquatic organisms.

#### Integration with other datasets

The MSAIRHD dataset can be combined with other regional datasets to investigate:

Land use change impacts on hydrological processes (by integrating with historical land use/land cover data).

Climate-hydrology relationships (by combining with gridded meteorological products such as *Peng et al*.^54^, *Qin et al*.^55^); reservoir and dam impacts (using the reservoir dataset by *Shen et al*.^56^); water quality dynamics (using the water quality dataset by *Lin et al*.^57^); soil moisture and evapotranspiration relationships (using products from *Li et al*.^58^; *Niu et al*.^59^).

#### Large-sample hydrological studies

MSAIRHD contributes to global hydrological research by providing data from a previously underrepresented region, enabling: continental and global hydrological model intercomparisons; understanding of hydrological diversity across different climate regimes and geographical regions; development and testing of hydrological theories under diverse conditions; improved understanding of global water cycle dynamics

### Data limitations and uncertainty

Users should be aware of the following limitations when utilizing the MSAIRHD dataset:

#### Data coverage

The dataset spans the period from 1958 to 1987, with no records available for 1986. The number of available stations varies across the upper reaches of the international rivers. For streamflow, data were available from 12 stations in the Irrawaddy Basin, 13 in the Salween Basin, 34 in the Mekong Basin, and 28 in the Red River Basin. Water level records were available from 14 Irrawaddy stations, 20 Salween stations, 49 Mekong stations, and 35 Red River stations. Sediment concentration data were available from 5 Irrawaddy stations, 7 Salween stations, 16 Mekong stations, and 12 Red River stations.

#### Quality variations

Data quality varies across stations and basins. Stations with longer records and continuous data provide more reliable estimates. Users should consult the quality metrics provided in the station catalogue.

#### Data gap limitations

While we retained stations with at least 1 consecutive year of data to maximize coverage, users conducting trend analyses should select stations with sufficient temporal continuity.

#### Transboundary considerations

The dataset only covers the upper reaches within Chinese territory. Downstream hydrological conditions may differ due to tributary contributions, and water management decisions in other countries.

#### Data sharing restrictions

Due to data-sharing restrictions stipulated by the providers, the original station observations cannot be disseminated. The dataset provides derived hydrological indices and metadata.

### Integration with global datasets

MSAIRHD helps to address the gap in large-sample global hydrological studies^7^ by contributing representative data from a previously underrepresented region. The dataset is complementary to:

The Caravan global community dataset^7,16,19^, the Global Streamflow Indices and Metadata Archive (e.g., *Chen et al*.^19^ and Do *et al*.^47^), the Global Runoff Data Centre (GRDC)^60^ global database; Regional CAMELS datasets (e.g., CAMELS-IND; *Mangukiya et al*.^31^; *Jiao et al*.^38^); the 74 hydrological indices in MSAIRHD are calculated using standardized definitions consistent with global datasets (*Chen et al*.^19^), enabling meaningful cross-regional comparisons and global-scale synthesis.

### Data access

The Mainland Southeast Asia International River Upper Reach Hydrological Dataset (MSAIRHD) is available at: Science Data Bank^49^. The dataset includes: station catalogue file (MSAIRHD_station_catalogue.xlsx). Hydrological indices file (International_River_Hydrological_Indices.xlsx). Available years for each station and the number of available stations per year in each basin.zip; python processing package (International_River_Hydrological_Indices.py). The hardcopy of the International Hydrological Yearbook of Tibet and Yunnan in the People’s Republic of China (http://www.mwr.gov.cn/english/) can be found in the National Library of China. Raw hydrological data supporting the findings of this study are also available from the corresponding author upon reasonable request.

## Data Availability

The Mainland Southeast Asia International River Upper Reach Hydrological Dataset (MSAIRHD) described in this paper is available during the submission process. The dataset primarily consists of XLSX-format files, including:(1) A station catalogue file named *MSAIRHD_station_catalogue.xlsx*. This file contains key metadata for each monitoring station, such as station name, sub-basin identifier, station ID, river name, longitude, latitude, drainage area (square kilometer), information regarding any station relocation, and multi-year average value of hydrological indices. The availability of streamflow, water level, and sediment data for each station is indicated by a “Y” in the respective column, with full details provided in Table 2. (2) Hydrological indices for each station data file named *Hydrological_Indices.xlsx*, containing the corresponding annual values for all calculated streamflow, water level, and sediment indices. (3) A Python package named *International_River_Hydrological_Indices.py* for computing the hydrological indices. This hydrological indices data can be obtained from https://doi.org/10.57760/sciencedb.36675, Cheng *et al*.^49^.

## Code availability

All codes for processing the international River hydrological indices are available at: https://github.com/Chengqp2022/International_River_Hydrological_Indices (adapted for MSAIRHD applications).
